# Chromosome Bridges Maintain Kinetochore-Microtubule Attachment throughout Mitosis and Rarely Break during Anaphase

**DOI:** 10.1371/journal.pone.0147420

**Published:** 2016-01-19

**Authors:** Judit Pampalona, Emanuele Roscioli, William T. Silkworth, Brent Bowden, Anna Genescà, Laura Tusell, Daniela Cimini

**Affiliations:** 1 Department of Biological Sciences, Virginia Tech, Blacksburg, VA 24061, United States of America; 2 Department of Cell Biology, Physiology and Immunology, Universitat Autònoma de Barcelona, Bellaterra, E-08193, Spain; 3 Biocomplexity Institute of Virginia Tech, Blacksburg, VA 24061, United States of America; Florida State University, UNITED STATES

## Abstract

Accurate chromosome segregation during cell division is essential to maintain genome stability, and chromosome segregation errors are causally linked to genetic disorders and cancer. An anaphase chromosome bridge is a particular chromosome segregation error observed in cells that enter mitosis with fused chromosomes/sister chromatids. The widely accepted Breakage/Fusion/Bridge cycle model proposes that anaphase chromosome bridges break during mitosis to generate chromosome ends that will fuse during the following cell cycle, thus forming new bridges that will break, and so on. However, various studies have also shown a link between chromosome bridges and aneuploidy and/or polyploidy. In this study, we investigated the behavior and properties of chromosome bridges during mitosis, with the idea to gain insight into the potential mechanism underlying chromosome bridge-induced aneuploidy. We find that only a small number of chromosome bridges break during anaphase, whereas the rest persist through mitosis into the subsequent cell cycle. We also find that the microtubule bundles (k-fibers) bound to bridge kinetochores are not prone to breakage/detachment, thus supporting the conclusion that k-fiber detachment is not the cause of chromosome bridge-induced aneuploidy. Instead, our data suggest that while the microtubules bound to the kinetochores of normally segregating chromosomes shorten substantially during anaphase, the k-fibers bound to bridge kinetochores shorten only slightly, and may even lengthen, during anaphase. This causes some of the bridge kinetochores/chromosomes to lag behind in a position that is proximal to the cell/spindle equator and may cause the bridged chromosomes to be segregated into the same daughter nucleus or to form a micronucleus.

## Introduction

Accurate chromosome segregation during mitosis is critical for the maintenance of genome integrity through subsequent generations. The products of DNA replication are held together from S-phase until mitotic entry, when they become visible as individual chromosomes, each constituted of two sister chromatids. Binding of sister chromatids, via kinetochores, to microtubules of the mitotic spindle is required for chromosome segregation. However, mitotic chromosomes must undergo several different changes before sister chromatid segregation can occur in anaphase. First, the chromosomes must condense. Moreover, the enzyme topoisomerase II must decatenate the two DNA molecules [1, 2] that persist in a catenated (tangled) state after DNA synthesis due to the intrinsic DNA topology. Finally, the sister chromatids are held together by cohesin complexes [3, 4] that must be removed at the metaphase-anaphase transition [5, 6] to allow for sister chromatid separation and segregation to opposite spindle poles. Defects in any of these processes generate chromosomes whose chromatids cannot separate from each other and produce a typical cellular phenotype, which is the presence of anaphase chromosome bridges. Indeed, cells treated with topoisomerase II inhibitors display high frequencies of chromosome bridges, and in some cases severe impairment of sister chromatid separation that results in complete failure of cell division [1, 7–9]. Similarly, defects in cohesin degradation interfere with anaphase chromosome segregation and can result in impaired cell division [6, 10, 11]. Chromosome bridges can also arise as a result of DNA repair-triggered chromosome fusion. For example, the DNA repair machinery repairs double strand breaks (DSB) by searching for neighboring DNA molecules to re-join the broken end(s) [12–14]. If the process occurs in G2 and the sister chromatid presents a DSB as well, then the two sister chromatids will likely be fused [15, 16]. Such fused sister chromatids will be unable to separate in anaphase, and will therefore form a bridge spanning the spindle midzone. If the DNA damage induces a DSB only in one of the two sisters or if the damage occurs prior to DNA replication, then the DNA repair machinery can induce fusion between different broken chromosomes [17]. In this case, the fused chromosomes can again form bridges spanning the spindle midzone. DSBs can be caused by a number of DNA damaging agents, including ionizing radiation, radiomimetic compounds, such as neocarzinostatin and bleomycin, and topoisomerase I and II inhibitors. Finally, certain defects in telomere structure can be recognized by the DNA repair machinery as DSBs. Indeed, both defects in telomere-associated proteins and excessive telomere shortening result in recruitment of DNA damage response proteins at uncapped chromosomes [18–20]. Current models suggest that dysfunctional telomeres are recognized as DSBs and are joined to other uncapped ends. This would explain the high frequencies of chromosome bridges in cells experiencing telomere attrition [21–23]. The end-to-end fusions observed in such cells include both fusions between sister chromatids and fusions between different chromosomes [24].

The classical model of chromosome bridge behavior during cell division is the Breakage/ Fusion/ Bridge (B/F/B) cycle, according to which broken chromosomes fuse with other broken chromosomes. The fused chromosomes will generate a bridge during mitosis, at which time the bridge will break to generate new broken ends, which will then fuse again with other broken ends during the following cell cycle [25, 26]. Thus, the B/F/B cycle could go on for prolonged periods of time leading to extensive rearrangements of the genome. Strikingly, in addition to structural chromosome aberrations, topoisomerase II inhibition can result in aneuploidy [7]. Similarly, telomere dysfunction was shown to be associated with aneuploidy [27, 28] and polyploidy [22, 29]. It was previously suggested, but never demonstrated, that telomere dysfunction-associated aneuploidy may arise via loss of attachment between the bridged chromosomes and the microtubules of the mitotic spindle [27, 28, 30]. In this study, we performed an in-depth analysis of individual chromosome bridges to gain insight into the potential mechanism underlying chromosome bridge-mediated aneuploidy. To this aim, we used a number of different strategies. First, we used p16-silenced human mammary epithelial cells (referred to as variant HMECs or vHMECs) [22], in which high frequencies of chromosome bridges are known to arise due to telomere dysfunction. Second, we experimentally induced chromosome bridges in non-transformed PtK1 cells and in the HeLa cancer cell line by acute treatment with the DNA damaging agent bleomycin. We performed a number of quantitative experiments, including one set specifically focused on testing the hypothesis that chromosome bridges may lose microtubule attachment during anaphase.

## Results

### Most chromosome bridges do not break during anaphase

**Fig 1:**
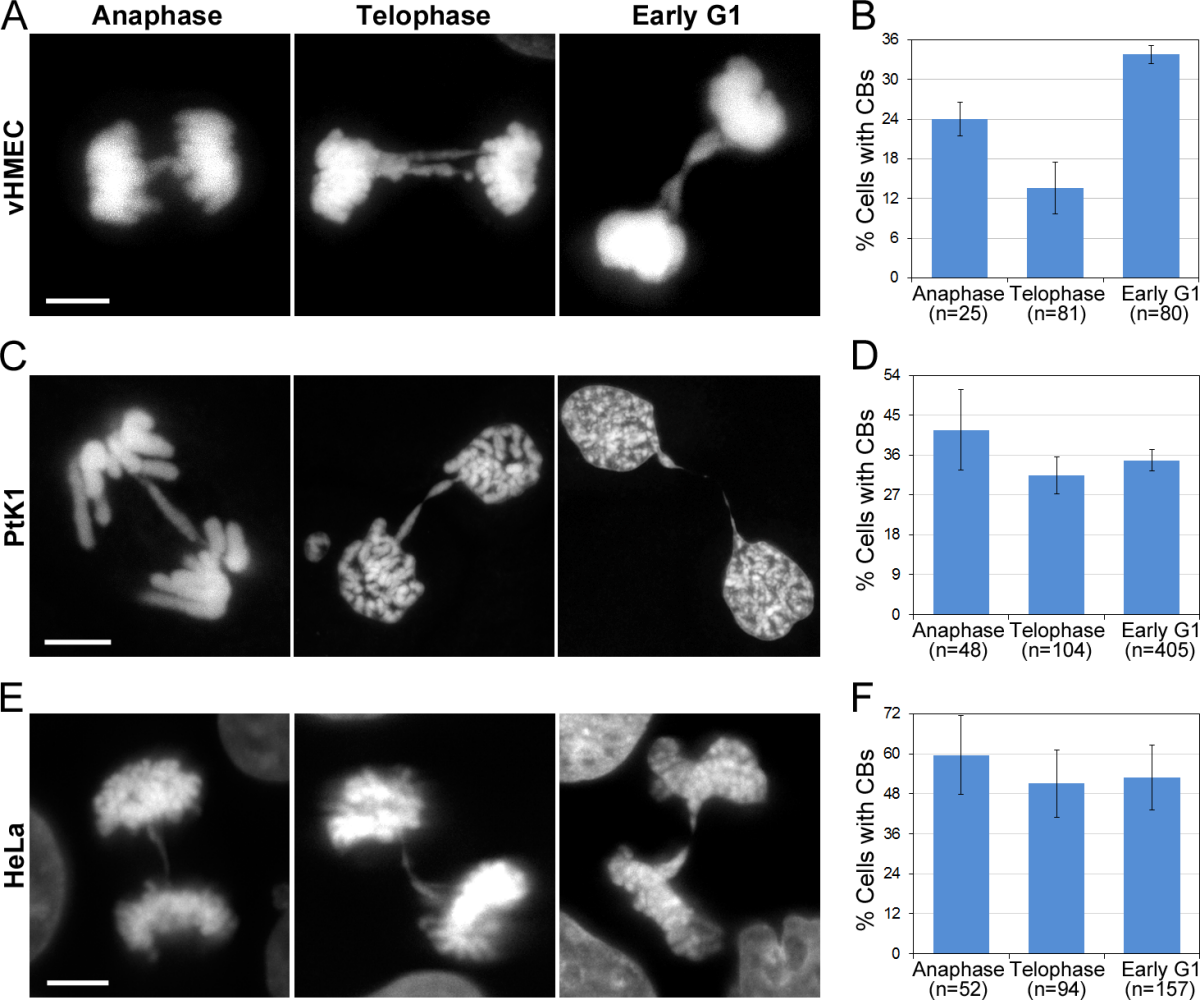
Many chromosome bridges do not break during anaphase. (A, C, E) Examples of chromosome bridges in vHMEC (A), PtK1 (C), and HeLa (E) cells at different mitotic stages. DAPI staining is shown for all cells. Scale bars, 5 μm. (B, D, F) Frequencies (mean ± s.e.) of bridges in vHMEC (B), PtK1 (D), and HeLa (F) cells. The reported n values represent the total number of cells analyzed from 2 (vHMECs) or 3 (PtK1 and HeLa) independent experiments.

The B/F/B cycle [25, 26] is widely accepted to explain the behavior of fused chromosomes. According to this model, chromosome bridges would undergo breakage during mitosis (likely during anaphase). We first sought to determine the stage of mitosis when chromosome bridge breakage occurs. To this end, we performed fixed-cell experiments in which we determined the frequencies of chromosome bridges at different stages of mitosis and at an early post-mitotic stage that we refer to as “early G1” (Fig 1). Early G1 cells were defined as cells that had completed mitosis, but in which the two daughter cells were still connected by a cytoplasmic bridge, as indicated by a residual microtubule mid-body structure connecting the two cells (not shown in the figure). If most bridges were breaking during anaphase, we would expect most of the chromosome bridges to disappear by telophase. Instead, we found that the fraction of telophase vHMECs exhibiting chromosome bridges was about 60% that of anaphase cells with bridges, indicating that only 40% of chromosome bridges are likely to break during anaphase (Fig 1A and 1B). Similar results were observed in bleomycin-treated PtK1 (Fig 1C and 1D) and HeLa cells (Fig 1E and 1F), in which an even smaller fraction of chromosome bridges disappeared between anaphase and telophase. In addition, analysis of “early G1” cells showed that in vHMECs, the frequency (33%) of early G1 cells with chromatin bridges was higher than the frequency of either telophase (17%) or anaphase (29%) cells with chromosome bridges (Fig 1B), suggesting that abscission may be significantly delayed in these vHMECs with chromosome bridges [31] compared to vHMECs at the same cell cycle stage, but without chromatin bridges. In PtK1 and HeLa cells, the chromosome bridges also persisted into early G1, but not to the extent observed in vHMECs. Despite this difference, the overall picture emerging from these data is that in animal cells many chromosome bridges do not break during anaphase/telophase, and instead persist well beyond completion of mitosis into early G1.

**Fig 2:**
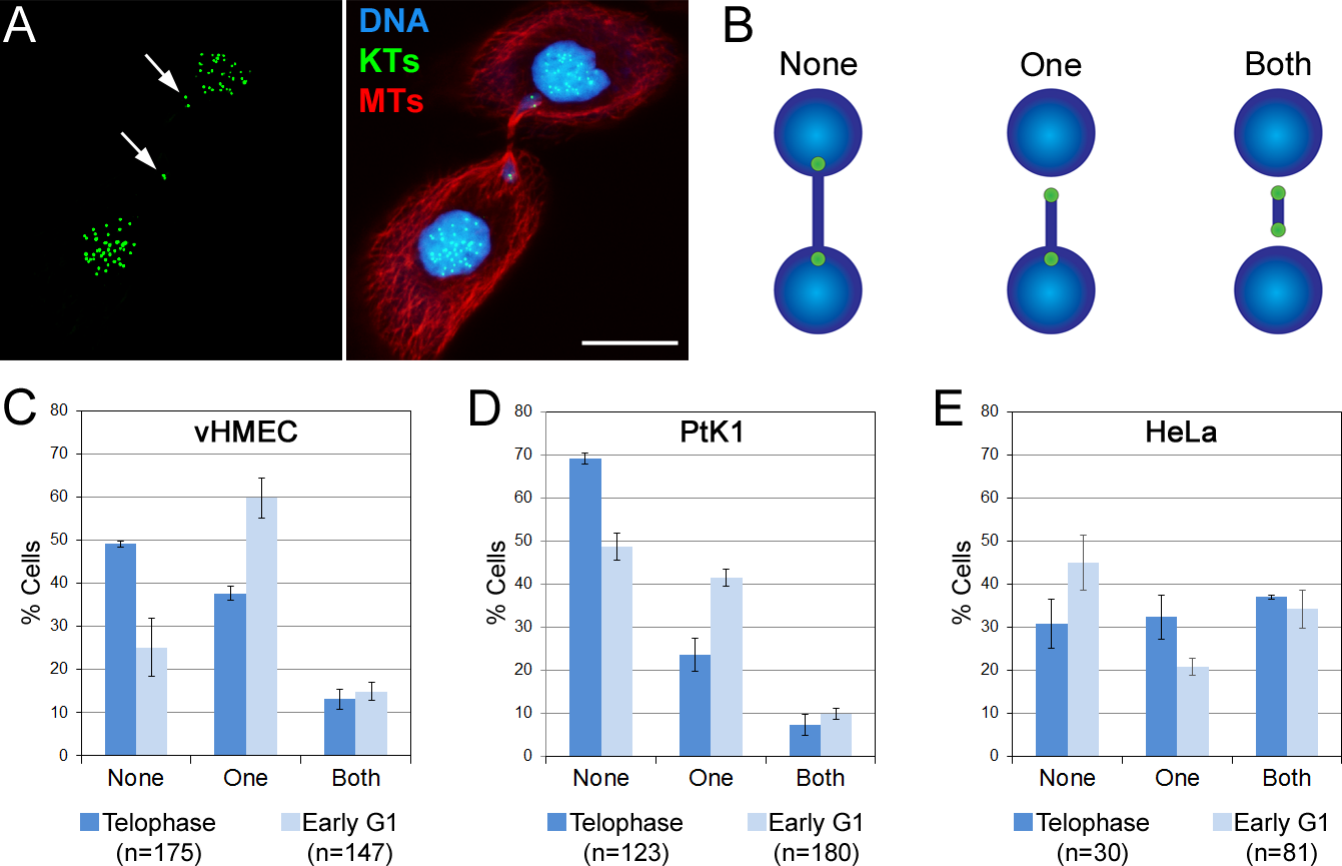
Chromosome bridges persist beyond completion of mitosis. (A) Example of early G1 vHMEC with a chromosome bridge whose ends are completely detached from the bulk of the chromatin in the daughter nuclei. Kinetochores (KTs, immunostained using CREST antibodies) are shown in green, microtubules (MTs) in red, and DNA in blue. Two KTs are visible at one end of the bridge and one KT at the other end (arrows). Scale bar, 10 μm. (B) Diagram illustrating how the bridges were classified. (C-E) Frequencies (mean ± s.e.) of bridges with KTs visible at both ends (Both), at one end (One), or not visible (None) because embedded in the bulk of the chromatin. The reported n values represent the total number of chromosome bridges analyzed from 2 independent experiments.

Further characterization of chromosome bridges at different cell cycle stages (Fig 2) also showed that as vHMECs and PtK1 cells progressed from telophase to early G1, the number of bridges in which we could detect the kinetochore at least at one end of the bridge increased substantially (Fig 2C and 2D). In other words, whereas in telophase cells ~50% (vHMEC) to ~70% (PtK1) of the bridges had kinetochores completely embedded in the two groups of segregated chromosomes, in early G1 cells these frequencies shifted, with a total of ~75% (vHMEC) and ~50% (PtK1) of the bridges displaying kinetochores that could be visualized at one or both ends of the bridge (Fig 2B, middle and right, and 2C-D). These results suggest that, when the two ends of the chromosome bridge become free to move at the end of mitosis due to mitotic spindle disassembly, these persistent chromosome bridges can produce tension that can cause the kinetochores at the end of the chromosome bridge to move away from the newly formed daughter nuclei, potentially resulting in the formation of micronuclei or abnormally shaped nuclei in the daughter cells [21, 28]. Although HeLa cells did not display the same stage-dependent changes observed in the other two cell lines, the number of cells with chromosome bridges displaying kinetochores at one or both ends was quite high (~70%) already in telophase. These observations further support our finding that many chromosome bridges do not break during anaphase in animal cells, in agreement with previous reports showing that chromosome bridges can persist well beyond completion of mitosis and delay abscission [32], or can cause cleavage furrow regression, and hence formation of binucleate/polyploid cells [29, 33]. Moreover, the persistence of unbroken chromosome bridges throughout mitosis may also play a role in telomere dysfunction-linked aneuploidy (see below) [27, 28, 34].

### Bridge kinetochores are shifted closer to the spindle equator compared to non-bridge kinetochores, but are not detached from their respective k-fibers

It was previously shown that chromosomes with critically shortened telomere ends are likely to fuse to each other and to appear in aneuploid numbers in the cell population [27, 28], either by segregation of the bridged chromosomes to one daughter nucleus or by inclusion of the bridged chromosome into a micronucleus [27].

**Fig 3:**
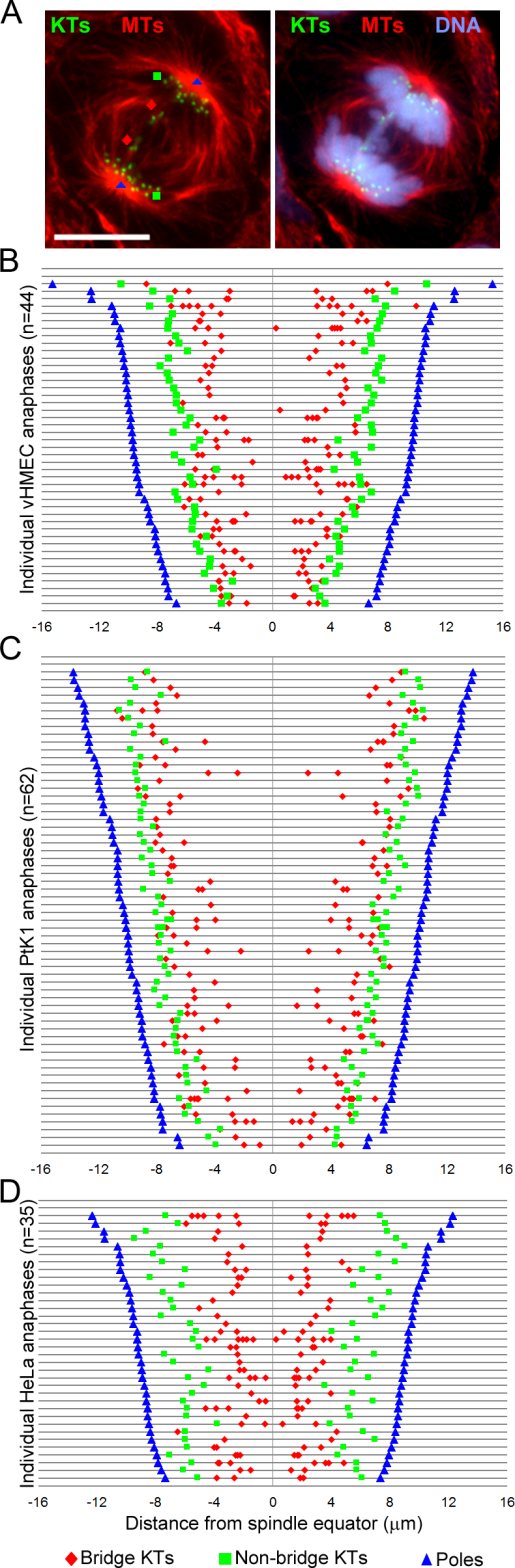
Bridge kinetochores are shifted close to the spindle equator compared to non-bridge kinetochores. (A) Example of anaphase vHMEC with chromosome bridge with staining for kinetochores (KTs, green, immunostained using CREST antibodies), microtubules (MTs, red), and DNA (blue). The symbols represent the location at which various elements of the mitotic apparatus were mapped. Blue triangle = position of spindle pole; green square = average position of 5 non-bridge KTs; red diamond = position of bridge KT. Scale bar, 10 μm. (B-D) Graphs displaying the relative distances of the various mapped elements from the spindle equator in vHMEC (B), PtK1 (C), and HeLa (D) cells. The position of the spindle equator was determined as the middle point between the two spindle poles. Each line in the graph represents an individual cell. For each cell, the position of the non-bridge KTs is reported as an average of five randomly selected KTs, whereas the bridge KT positions are reported individually. Thus, if a cell had multiple chromatin bridges, multiple pairs of red diamonds appear on the corresponding line. The reported n values represent the total number of cells analyzed from 2 independent experiments.

To gain insight into the mechanism by which fused chromosomes mis-segregate during mitosis, we mapped the position of bridge and non-bridge kinetochores with respect to the spindle equator within individual vHMEC, PtK1, and HeLa cells (as diagrammed in Fig 3A). We found that on average, the kinetochores of the chromosome bridge (bridge kinetochores, red diamonds in Fig 3B–3D) were positioned much closer to the spindle equator and farther away from the spindle poles (blue triangles in Fig 3B–3D) compared to the average position of kinetochores from normally segregating chromosomes (non-bridge kinetochores, green squares in Fig 3B–3D). The most noticeable results were observed in HeLa cells, but this is not surprising because the other two cell types were expected to possess longer bridges due to fusion at the telomeres (vHMECs) or large chromosome size (PtK1s). These data indicate that bridged chromosomes display different dynamics of anaphase poleward movement compared to the rest of the chromosomes, which causes the bridged chromosomes to persist in a position proximal to the spindle equator. This, in turn, can lead to mis-segregation, by either inclusion of the non-disjoint bridged chromosomes into the same daughter nucleus or by inclusion into a micronucleus (see Discussion for further detail).

**Fig 4:**
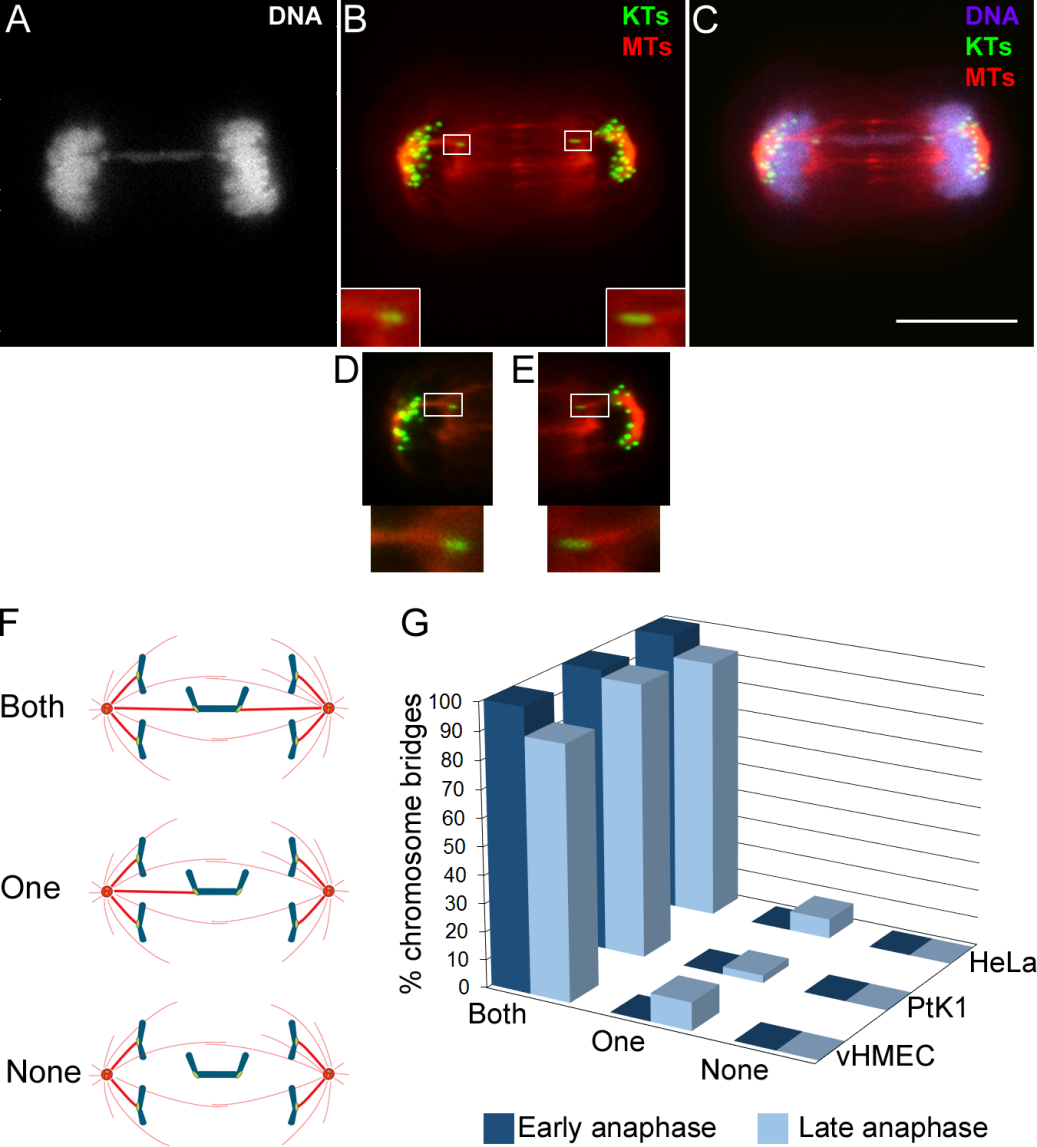
Nearly all bridge kinetochores are bound to k-fibers during anaphase. (A-C) Example of anaphase vHMEC stained for DNA (grey/blue), kinetochores (KTs, green, immunostained using CREST antibodies), and microtubules (MTs, red) and possessing a chromosome bridge. The overlay of KTs and MTs is shown in B and the overlay of all three colors is shown in C. The KT and MT images in (B) and (C) are maximum intensity projections of Z-stacks. The insets in B show enlarged (300%) views of the boxed regions. Scale bar, 10 μm. (D-E) Single focal planes of the left (D) and right (E) portions of the mitotic spindle that provide better visualization of the bridge KTs and their associated k-fibers. Enlarged (300%) views of the boxed regions are displayed at the bottom. (F) Diagram illustrating how KT-MT attachment (presence/absence of k-fibers) was classified in anaphase cells. To ensure unbiased evaluation, the presence of a k-fiber was determined by background-corrected fluorescence intensity quantification of α-tubulin immunostaining (see materials and methods section for details on fluorescence intensity quantification). (G) Frequencies of chromosome bridges with two (both), one, or no (none) k-fibers. The data represent the average from 2 independent experiments in which a total of 86 (vHMEC), 92 (PtK1), or 22 (HeLa), chromosome bridges were analyzed.

It could be argued that the shifted position of bridge kinetochores towards the spindle equator may be the result of detachment of kinetochore microtubules caused by pulling forces exerted by the intervening chromatin, as previously hypothesized [27, 28, 30]. To test this hypothesis, we studied the kinetochore-microtubule attachment in anaphase bridges occurring in vHMECs or in bleomycin-treated PtK1 and HeLa cells, and analyzed the presence of k-fibers at the kinetochore interface by tubulin fluorescence quantification (Fig 4A–4F; see materials and methods for details). We discriminated cells in early anaphase (kinetochores located close to the poles, but no noticeable spindle elongation) from cells in late anaphase (prominent spindle elongation), and found that all chromosome bridges exhibited kinetochore-microtubule attachments during early anaphase, and nearly all bridges in late anaphase cells had both kinetochores from the same bridge attached to microtubules (Fig 4G). Of the very few chromosome bridges we found with no obvious k-fibers at one end, most exhibited a stretched kinetochore appearance with kinetochores positioned at opposite ends, suggesting that microtubule attachment had been present, but either the k-fiber had disassembled immediately before or during fixation, or it was not thick enough for identification based on our fluorescence quantification criteria (see materials and methods for details), but still present. In a few cases (2 out of 86 in vHMECs) of bridges with k-fibers only on one side, the unattached kinetochore did not display obvious stretch and was clearly shifted to the opposite pole, suggesting the possibility that the unattached half of the bridged chromosome was simply being dragged towards the opposite pole by the attached half of the bridge as a result of either kinetochore attachment failure or premature k-fiber detachment. These results suggest that only in rare cases, during the segregation of a chromosome bridge, the forces exerted by the intervening chromatin may cause k-fiber detachment. Rather, such forces may cause stabilization of the kinetochore-microtubule attachments [35], resulting in “hyperstable” attachments that will be difficult to break and will be maintained until the time of spindle disassembly.

### K-fibers bound to bridge kinetochores can elongate during anaphase

**Fig 5:**
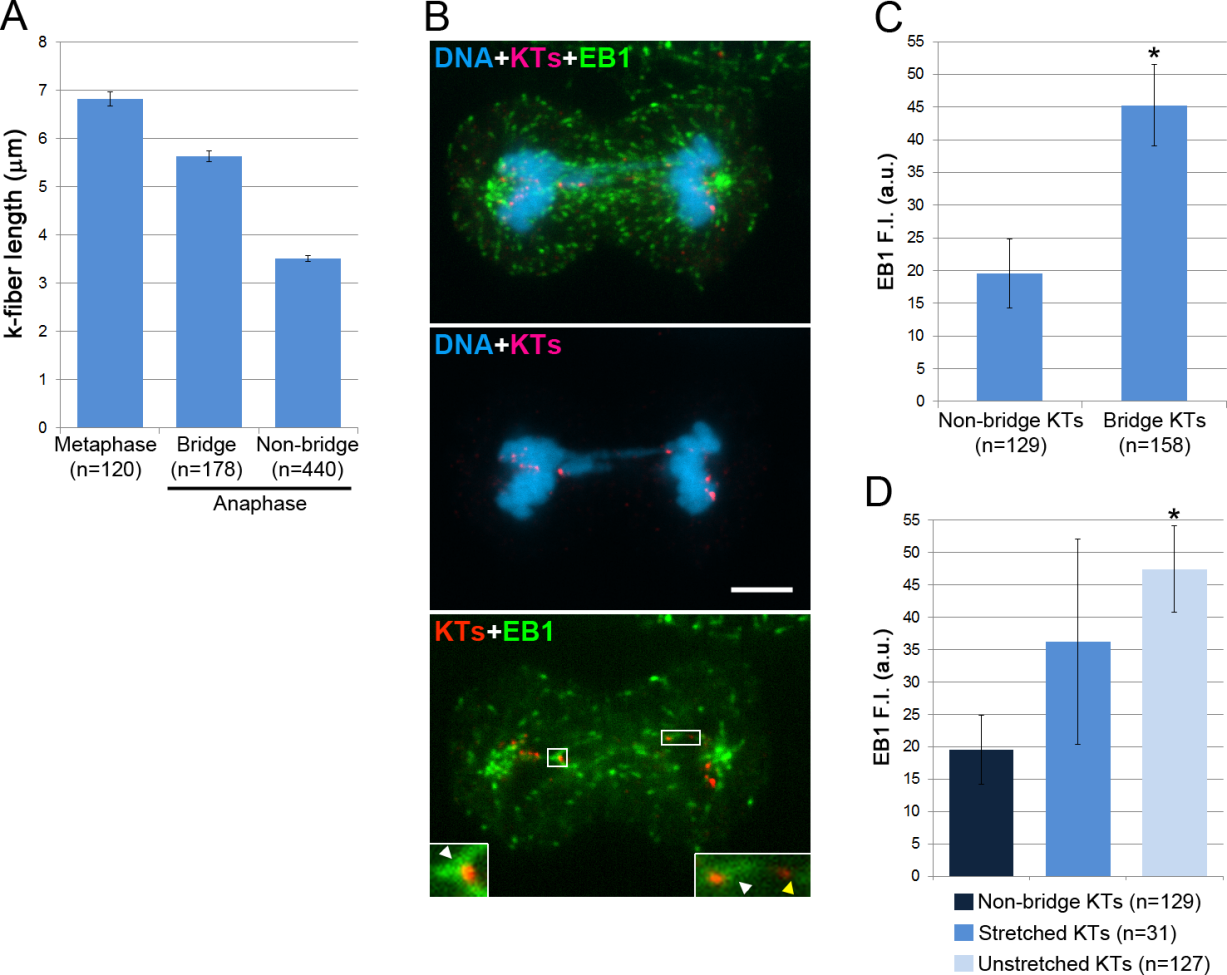
Bridge k-fibers do not display typical anaphase behavior. (A) Average metaphase k-fiber length and anaphase k-fiber length for bridge and non-bridge kinetochores (KTs) in vHMECs. Note that whereas the non-bridge k-fibers shorten by nearly 50% in anaphase, the bridge k-fibers exhibit a very modest length change. (B) Example of chromosome bridge in an anaphase vHMEC immunostained for DNA (blue), kinetochores (KTs, red, immunostained using CREST antibodies), and EB1 (green). The top image shows an overlay of maximum intensity projections of Z-stacks of immunostained KTs and EB1 with a single focal plane image of DAPI-stained DNA. The middle and bottom images display overlays of single focal planes corresponding to the focal plane including the bridge KTs. The insets in the bottom image display enlarged (300%) views of the boxed regions and include the bridge KTs as well as a non-bridge KT. White arrowheads point at the EB1 signal associated with the bridge KTs, whereas the yellow arrowhead points at the MT face of the non-bridge KT and illustrates the low level of EB1 labeling. Scale bar, 10 μm. (C) Average EB1 background-corrected fluorescence intensity (F.I.) at bridge and non-bridge KTs. (D) Average EB1 F.I. at non-bridge KTs vs. stretched and unstretched bridge KTs. For both (C) and (D), the asterisk denotes statistically significant difference (t-test, p<0.01) when data were compared to data from non-bridge KTs. The reported n values represent the total number of KTs analyzed from 2 independent experiments.

A mechanism that would be alternative to k-fiber detachment and that could explain the shifted position of the bridge kinetochores close to the spindle equator is the lengthening of the bridge k-fibers during anaphase. To test this hypothesis, we used vHMECs because in these cells the total chromosome number is not as large as in HeLa cells (important for feasibility of EB1 quantification experiments described below) and chromosome size is not as large as in PtK1 cells (which may result in a more pronounced bridge-dependent generation of tension at the kinetochore-microtubule interface of vHMECs). First, we measured the average k-fiber length in metaphase vHMECs, and compared it to the average bridge k-fiber length in anaphase. We found that, although k-fibers from chromosome bridges on average were not longer than metaphase k-fibers, they did not shorten as much as non-bridge k-fibers (Fig 5A) as cells progressed from metaphase to anaphase. This suggested that bridge k-fibers either shortened for a limited period of time and then stalled or lengthened slightly, or that they were alternating between periods of shortening and periods of lengthening during anaphase. To gain further insight, we quantified EB1 (a marker of microtubule plus-end polymerization) fluorescence (Fig 5B) at bridge vs. non-bridge kinetochores, and found that bridge kinetochores exhibited significantly higher EB1 fluorescence intensity compared to non-bridge kinetochores (Fig 5C). This result indicates that the microtubules bound to bridge kinetochores are more likely than non-bridge kinetochores to be in a polymerization state during anaphase.

We also observed that in about 20% of the bridges, one kinetochore appeared stretched (see example in Fig 4B, right bridge kinetochore). We reasoned that the appearance of bridge kinetochores as stretched or unstretched may depend on the efficiency at which the associated k-fiber polymerized during spindle elongation. Indeed, unstretched bridge kinetochores were found to exhibit on average higher EB1 fluorescence intensity compared to stretched bridge kinetochores (Fig 5D). These data suggest that the stretched appearance of certain bridge kinetochores can be explained by insufficient polymerization of the associated k-fiber during anaphase in response to the tension generated by the bridge. It could also be possible that the bridge k-fibers alternate between periods of shortening (at which times the DNA may become stretched) and periods of lengthening (at which time no further DNA stretching would occur).

## Discussion

### Large fractions of chromosome bridges do not break during anaphase

Our data showed that most chromosome bridges that can be visualized in mid-late anaphase persist throughout mitosis and into early G1. Because the DNA is highly condensed in mitosis, it is not surprising that some anaphase chromosome bridges may get stretched without breaking. Indeed, micromanipulation studies showed that isolated newt chromosomes can be extended up to about 80 times their original length without breaking [36] and breakage occurs when chromosomes are stretched about 100-fold their original length with an applied force of the order of 100 nN [37]. Similar findings were reported for human chromosomes [38]. The amount of stretching for anaphase chromosome bridges will be determined by the extent of spindle elongation and/or the forces that the spindle can produce, as well as the length of the intervening chromatin. The maximum force that the spindle can exert on individual anaphase chromosomes has been estimated in grasshopper spermatocytes to be approximately 700 pN [39, 40], which is substantially lower than the force necessary to stretch a chromosome to the point of breakage [37]. Furthermore, by the end of mitosis the mitotic spindle reaches lengths that are not more than ~2-fold the average metaphase spindle length (e.g., 12.8 ± 3.3 μm in metaphase vs. 24.5 ± 3.9 μm at the end of mitosis in vHMECs). Thus, whereas spindle elongation may account for some of the stretching observed for anaphase chromosome bridges, it does not produce the type of forces and the amount of stretching necessary to break the chromatin bridge. Moreover, tension generated at bridge kinetochores may result in kinetochore microtubule polymerization, thus further attenuating the stretching caused by anaphase spindle elongation. Support for such a mechanism is provided by our EB1 quantifications (Fig 5), and it is in agreement with both the idea that tension at the kinetochore directly influences the polymerization/depolymerization state of kinetochore microtubules [35] and with the previously observed lengthening of k-fibers bound to merotelically attached anaphase lagging chromosomes [41].

What then induces some chromosome bridges to break? The most likely scenario is that chromosome and spindle mechanics act in concert to cause bridge breakage. For example, live-cell studies showed that acentric chromosome fragments in insect spermatocytes are transported poleward during anaphase most likely due to microtubule poleward flux [42, 43]. Similarly, in plant endosperm cells acentric chromosome fragments are pulled poleward at the time of phragmoplast formation [44–46] via a kinetochore-independent mechanism [45]. It is possible that microtubule poleward flux or other microtubule-dependent forces may similarly act on chromosome bridges, thus causing them to stretch poleward and eventually break. This phenomenon could explain the prevalence of bridge breakage in plant cells [26] as opposed to mammalian tissue culture cells (this study). Indeed, poleward movement of acentric fragments in vertebrate somatic cells has not been reported, and acentric chromosome fragments are instead believed to lag behind at the spindle equator during anaphase [47, 48]. What other forces could account for bridge breakage in vertebrate somatic cells? A recent study reported that maximum chromosome compaction in mammalian tissue culture cells is achieved in late anaphase [49]. Such chromosome condensation may cause some regions of the chromosome bridge to become stretched and break as other regions attempt to undergo anaphase compaction. Moreover, a study in yeast [50] described a phenomenon termed “adaptive hypercondensation” [50, 51], which induces enhanced condensation of chromosome arms spanning the spindle midzone via an Aurora B kinase-dependent mechanism [50]. Although this phenomenon has not been described in metazoans, it is possible that a similar mechanism may be activated in the presence of chromosome bridges, thus inducing breakage of a fraction of chromosome bridges during ana/telophase. Alternatively, some regions of the chromosome may be more easily stretched and broken due to intrinsic structural features (see for example [52–54]). Our observations also suggest that if any bridge breakage occurs due to mechanical stress in mammalian cells, this must happen mostly in anaphase, given that when chromosome bridges persist long enough they are most likely to result in cleavage furrow regression or delayed abscission [31]. Nevertheless, we cannot exclude that bridges persisting into cytokinesis may also break during cleavage furrow ingression due to mechanical stress imposed by the ingressing furrow on the chromosome bridge, as previously suggested in other studies [55, 56] or via a biochemical pathway linked to cytokinesis/abscission.

### Chromosome bridges, KT-MT attachment, and aneuploidy

**Fig 6:**
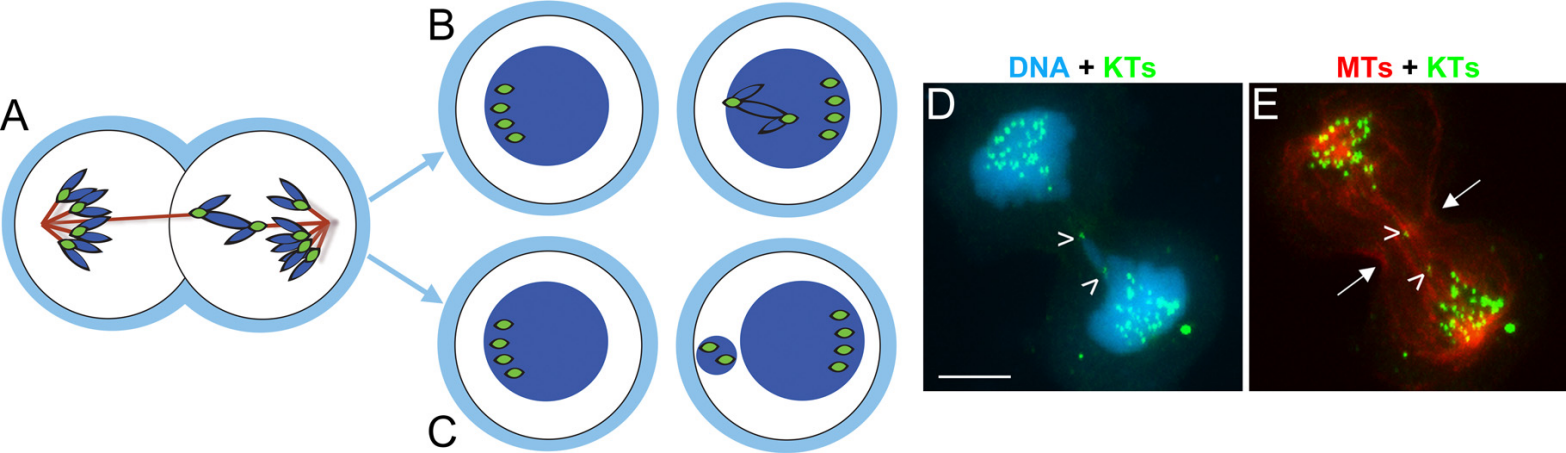
Differential shortening/lengthening of k-fibers during anaphase can lead to segregation of the bridged chromosomes to the same daughter cell. (A-C) Diagram illustrating how differential shortening/lengthening of k-fibers during anaphase would lead to segregation of the bridged chromosomes to the same daughter cell. Depending on the extent of the length differential between the two k-fibers bound to the bridge kinetochores, the bridged chromosome may end up in the main nucleus (B) or in a micronucleus (C). (D-E) Example of vHMEC in which the bridged chromosomes are segregating to the same daughter cell. DNA is shown in blue, kinetochores (KTs, immunostained using CREST antibodies) in green, and microtubules (MTs) in red. Open arrowheads point to the two ends/KTs of the chromosome bridge. Note that the cleavage furrow (arrows) is ingressing on one side of the chromosome bridge, thus pushing the whole bridge into one of the daughter cells. Scale bar, 5 μm.

Several studies have previously identified some degree of aneuploidy under conditions that would be expected to induce chromosome structural aberrations, and hence chromosome bridges as a main defect. For example, chemical inhibition of topoisomerase II to a level that induces high frequencies of anaphase chromosome bridges, also results in both chromosome breakage and aneuploidy [7]. Moreover, certain cancer cell types exhibit high frequencies of both anaphase chromosome bridges and aneuploidy [28, 57]. Finally, in vHMECs the chromosomes with the shortest telomeres are frequently found in aneuploid numbers within the cell population [27]. It was previously hypothesized that this could be a consequence of bridge kinetochore detachment from spindle microtubules [27, 28, 30]. However, this hypothesis was never tested before. We now show that bridge kinetochores very rarely, if ever, lose their attachment to spindle microtubules (Fig 4), and the ends of the chromosome bridge only become free to move upon spindle disassembly (Fig 2). Instead, we present evidence (Fig 5) for a mechanism in which the k-fibers bound to bridge kinetochores do not significantly shorten, and possibly elongate, during anaphase. If the two k-fibers change length differentially (e.g., one shortens and the other lengthens; Fig 6A), then the bridged chromosomes would segregate to the same daughter cell (Fig 6B and 6C). Whether the bridged chromosome ends up in the main nucleus (Fig 6B) or in a micronucleus (Fig 6C) may simply depend on the extent of the length differential between the two k-fibers bound to the bridge kinetochores. In a few cells (see Fig 3 and example in Fig 6D and 6E) we found evidence of differential k-fiber lengthening. However, it is possible that such segregation of bridged chromosomes to the same daughter cell may be hard to visualize if it occurs early in anaphase, when the arms of many chromosomes still span the spindle midzone, thus making the identification of this type of segregation very challenging. It should be noted that if this type of segregation occurred in the case of an inter-chromatid bridge, it would result in aneuploidy, thus explaining the numerous reports indicating that defects normally expected to result in chromosome rearrangements due to the B/F/B cycle are also associated with aneuploidy. Moreover, segregation of a chromosome bridge into a micronucleus may result in both aneuploidy (there may be a chromosome loss in the cell without the micronucleus) and accumulation of DNA damage within the micronucleus itself [58, 59].

### Conclusion

The B/F/B cycle is a widely accepted model, which has become the textbook explanation of how chromosome bridges may lead to extensive genome rearrangements. However, whereas the B/F/B cycle may fully explain what happens in certain cell types (e.g., plant cells and insect spermatocytes), such a model may underestimate the effect of anaphase chromosome bridges on the karyotype of animal somatic cells. Indeed, it appears that in animal somatic cells a chromosome bridge may lead to a number of different outcomes, including chromosome breakage, polyploidy (by cleavage furrow regression), aneuploidy, and possibly cell cycle arrest (by abscission checkpoint activation). Given the myriad of possible outcomes anaphase chromosome bridges can produce, future studies should be aimed at elucidating what determines a bridge to break, mis-segregate, or inhibit cytokinesis.

One could argue that the multiple fates of chromosome bridges may explain the complex karyotypes of cancer cells, in which high rates of both aneuploidy and chromosome rearrangements are observed. However, elevated frequencies of chromosome bridges have only been reported for certain specific cancer types [60–63], whereas in most other cancer cells anaphase lagging chromosomes appear to be a far more common chromosome segregation defect [64–68]. Thus, it is possible that chromosome bridges contribute to tumor initiation, when chromosome rearrangements and aneuploidy may initially arise, whereas once telomeres become stabilized by re-activation of telomere maintenance mechanisms, the major contributors to chromosomal instability may be spindle multipolarity and anaphase lagging chromosomes [69].

## Materials and Methods

### Cell culture conditions and treatment

p16-silenced human mammary epithelial cells (vHMECs) were obtained from Cell Applications Inc. (San Diego, CA); *Potorous tridactylus* kidney epithelial cells (PtK1 cells) and HeLa cells were gifts of Ted Salmon (University of North Carolina at Chapel Hill). vHMECs were cultured in MEGM medium (BioWhittaker Inc., Walkersville, MD) supplemented with epidermal growth factor, insulin, hydrocortisone, gentamycin/amphotericin-B, and bovine pituitary extract. PtK1 cells were cultured in Ham’s F12 (Sigma Chemical Co., St. Louis, MO) supplemented with 10% fetal bovine serum, antibiotics and antimycotics. HeLa cells were cultured in DMEM (Gibco-Thermo Fisher, Waltham, MA) supplemented with 10% fetal bovine serum, antibiotics and antimycotics. All cells were maintained at 37°C, 5% CO_2_, in a humidified incubator. For experiments, cells were plated on sterilized acid-washed coverslips inside sterile 35 mm Petri dishes.

To experimentally induce anaphase chromosome bridges in PtK1 and HeLa cells, Bleocin^TM^ (antibiotic from *Streptomyces verticillus*; Calbiochem, San Diego, CA) was added to exponentially growing cells at a final concentration of 60 μg/ml, and incubated for 3 hours at 37°C, 5% CO_2_, in a humidified atmosphere. After washing out the drug, cells were re-incubated in fresh media for 24 hours before fixation.

### Fixation and immunostaining

vHMECs were briefly rinsed in 1x PBS, pre-fixed in 4% formaldehyde for 10 sec, permeabilized for 5 min in a solution of 1x PHEM containing 0.5% Triton X-100, fixed in 4% formaldehyde for 20 min, and finally washed 3 times for 5 min in PBS. PtK1 and HeLa cells were first rinsed in PBS and fixed in an ice-cold solution of 95% methanol and 5 mM EGTA for 5 min at room temperature, followed by further fixation in 95% methanol/5 mM EGTA mixture for 20 min at -20°C. The subsequent steps were the same for all cell types. Cells were washed in PBS and blocked in 10% boiled goat serum (BGS) for 1 h at room temperature and then incubated in primary antibodies diluted in 5% BGS, overnight at 4°C. The coverslips were then washed 3 times in PBST (PBS with 0.05%Tween-20) and incubated in secondary antibodies in 5% BGS for 45 min at room temperature. Primary antibodies against kinetochores (Anticentromere Antibodies, derived from human CREST patient serum; Antibodies Inc., Davis, CA), microtubules (DM1A, mouse-anti-α-tubulin; Sigma-Aldrich, Raleigh, NC), and EB1 (mouse-anti-EB1; BD-Biosciences, Franklin Lakes, NJ) were diluted 1:100, 1:500, and 1:100, respectively. Secondary antibodies X-Rhodamine goat-anti-human (AbCam, Cambridge, UK) and Cy5 goat-anti-mouse (Jackson ImmunoResearch Laboratories Inc., West Grove, PA) were both diluted 1:100. DNA was counterstained with 1 μM YoPro (Invitrogen, Carlsbad, CA) diluted in PBST, for 5 min. Coverslips were mounted in an antifade solution containing 90% glycerol and 0.5% N-propyl gallate.

### Image acquisition and analysis

Immunostained cells were imaged using a Nikon Eclipse TE2000-U inverted microscope equipped with a Swept Field Confocal system (Prairie Technologies Inc., Middleton, WI) and HQ2 CCD camera (Photometrics, Tucson, AZ). The confocal head harbored a filter set for illumination at 488 nm, 568 nm and 647 nm wavelengths through a 400 mW argon laser and a 150 mW krypton laser. All components were under the control of NIS elements software (Nikon Instruments Inc., Melville, NY) on a PC computer. Images were acquired using a 100x 1.4 NA Plan-Apochromatic phase–contrast objective lens. Z-stacks were acquired at 0.6 μm steps through each cell.

Fluorescence intensity measurements with background subtraction of both α-tubulin and EB1 immunostaining were performed on individual focal planes using the NIS elements software. For α-tubulin fluorescence intensity measurements, a square region of interest measuring 0.1 μm^2^ was drawn on the microtubule side of the microtubule-kinetochore interface and the mean fluorescence intensity within this region was automatically measured and recorded by the software. The mean background fluorescence intensity was measured in two smaller regions of interest (0.05 μm^2^ each) placed laterally and adjacent to both sides of the 0.1 μm^2^ region of interest. These background measurements were then averaged and subtracted from the fluorescence intensity measured within the 0.1 μm^2^ region. The value obtained represented the background-corrected fluorescence intensity of the k-fiber. A kinetochore was considered to be attached to a k-fiber when the background-corrected fluorescence intensity of the k-fiber in arbitrary units (a.u.) was >40. A similar method was used to quantify EB1 fluorescence intensity. The 0.1 μm^2^ square was traced in a region juxtaposed to the kinetochore on the side facing the spindle pole and the background fluorescence was measured in two 0.05 μm^2^ regions, one on each side of the 0.1 μm^2^ square. The average background fluorescence was then subtracted from the fluorescence intensity measured within the 0.1 μm^2^ region to obtain the background-corrected EB1 fluorescence intensity.

For kinetochore positioning data, actual 3-Dimensional distances between the two spindle poles (pole-pole distance) and between each kinetochore (for both bridge and non-bridge kinetochores) and the spindle pole that was closest to it (kinetochore-pole distance) were measured utilizing a standard function contained within the NIS elements image analysis package. The kinetochore-pole distance value corresponding to the non-bridge chromosomes was an average length from five different kinetochores within the same cell. Similarly, k-fiber length was measured in 3-D as the distance between a kinetochore and its closest pole.
